# CSF neurofilament light chain reflects corticospinal tract degeneration in ALS

**DOI:** 10.1002/acn3.212

**Published:** 2015-05-25

**Authors:** Ricarda A L Menke, Elizabeth Gray, Ching-Hua Lu, Jens Kuhle, Kevin Talbot, Andrea Malaspina, Martin R Turner

**Affiliations:** 1 Nuffield Department of Clinical Neurosciences, University of OxfordOxford, United Kingdom; 2 Centre for Neuroscience and Trauma, Blizard Institute, Barts and the London School of Medicine and Dentistry, Queen Mary University of LondonLondon, United Kingdom; 3 Department of Neurology, University Hospital BaselBasel, Switzerland

## Abstract

**Objective:**

Diffusion tensor imaging (DTI) is sensitive to white matter tract pathology. A core signature involving the corticospinal tracts (CSTs) has been identified in amyotrophic lateral sclerosis (ALS). Raised neurofilament light chain protein (NfL) in cerebrospinal fluid (CSF) is thought to reflect axonal damage in a range of neurological disorders. The relationship between these two measures was explored.

**Methods:**

CSF and serum NfL concentrations and DTI acquired at 3 Tesla on the same day were obtained from ALS patients (*n* = 25 CSF, 40 serum) and healthy, age-similar controls (*n* = 17 CSF, 25 serum). Within-group correlations between NfL and DTI measures of microstructural integrity in major white matter tracts (CSTs, superior longitudinal fasciculi [SLF], and corpus callosum) were performed using tract-based spatial statistics.

**Results:**

NfL levels were higher in patients compared to controls. CSF levels correlated with clinical upper motor neuron burden and rate of disease progression. Higher NfL levels were significantly associated with lower DTI fractional anisotropy and increased radial diffusivity in the CSTs of ALS patients, but not in controls.

**Interpretation:**

Elevated CSF and serum NfL is, in part, a result of CST degeneration in ALS. This highlights the wider potential for combining neurochemical and neuroimaging-based biomarkers in neurological disease.

## Introduction

Neurofilament in cerebrospinal fluid (CSF) and serum has emerged as a much-needed candidate biomarker for the fatal adult neurodegenerative disorder amyotrophic lateral sclerosis (ALS).^1^ The neurofilament light chain protein (NfL) is the main structural constituent of the neuroaxonal cytoskeleton and mainly found in large, myelinated axons. Elevated NfL CSF concentrations seem likely to reflect axonal loss. Increased CSF NfL has been previously reported in ALS,^2^–^4^ and levels found to correlate with measures of disease severity and progression in ALS.^5^,^6^

In addition to neurochemical markers, measures derived from noninvasive magnetic resonance diffusion tensor imaging (DTI), such as fractional anisotropy (FA) and radial diffusivity (RD) have been found to be sensitive to microstructural white matter alterations. While the former metric represents the degree of anisotropy of the diffusion of water molecules in axons and decreased FA is a frequent correlate of brain tissue injury, RD is a measure of diffusion perpendicular to the axon, and therefore commonly linked to the microstructure of myelin.^7^ Specifically, in ALS, a strong DTI white matter signature involving the corticospinal tracts (CSTs) and motor fibers in the corpus callosum (CC) has been consistently identified at group level,^8^,^9^ and FA and RD measures in the CSTs were found to correlate with upper motor neuron (UMN) score and progression rate.^10^,^11^

While numerous studies concluded that NfL or MRI measures show promise as biomarkers for diagnosis or as predictors of disease progression in different neurological disorders, to date there has been little attempt to directly relate the two.

Gaining insight in how and where in the brain these different measures relate to each other in health and disease may assist the development of improved, combined biomarker panels. Therefore, we investigated the relationship between CSF NfL levels and DTI measures of white matter microstructural integrity in a cohort of ALS patients and healthy controls, and furthermore attempted to correlate neurochemical data with clinical scores in the patient group. We repeated the analyses for serum NfL concentrations, for which there was a large cohort available with both measures.

## Methods

### Participants

Prevalent and incident cases of ALS from a large tertiary referral clinic were offered participation in Phase 1 of the Oxford Study for Biomarkers in MND (“BioMOx”) between 2009 and 2013. Ethical approval for all procedures was obtained in advance (South Central Oxford Ethics Committee: 08/H0605/85), with written informed consent obtained from all participants. All patients in this study were apparently sporadic, that is, reporting no family history of ALS or frontotemporal dementia (FTD), and diagnosed by one of two experienced neurologists (M. R. T., K. T.) according to standard criteria. The revised ALS Functional Rating Scale (ALSFRS-R) was used to assess disability on the day of study (M. R. T.). Disease duration was calculated from symptom onset to scan date in months, and the rate of progression was determined as (48 − ALSFRS-R)/disease duration. A clinical UMN clinical burden score of 15 comprised the number of pathologically brisk reflexes (abnormal glabellar tap response [1], facial jerk [1], jaw jerk [1], biceps [2], triceps [2], finger [2], knee [2], ankle [2] reflexes, and extensor plantar responses [2]).

### Determination of CSF and serum NfL concentrations

CSF and serum samples were processed within 1 h of extraction and on the same day as the MRI (M. R. T.). Samples were centrifuged, aliquoted, and stored in polypropylene tubes at −80°C until analysis. An electrochemiluminescence enzyme-linked immunosorbent assay (ELISA) was used to quantify NfL as previously described.^12^

### MRI acquisition

Scans were performed at the Oxford Centre for Clinical Magnetic Resonance (OCMR) using 3 T Siemens Trio scanner (Siemens AG, Erlangen, Germany) with a 12-channel head coil. For each subject T1-weighted images were obtained using a 3D Magnetization Prepared-Rapid Acquisition Gradient Echo (MP-RAGE) sequence (192 axial slices, flip angle: 8°, 1 × 1 × 1 mm^3^ voxel size, TE/TR/TI = 4.7/2040/900 msec). Acquisition time for the MP-RAGE image was 6 min. Whole-brain DTI images were acquired using an echoplanar imaging sequence (60 isotropic directions; *b* value = 1000 sec/mm^2^; echo time/repetition time = 94/10,000 msec; 2 × 2 × 2 mm^3^ voxel size; 65 slices). In addition, four images without diffusion weighting were acquired. Furthermore, a field map was acquired using a gradient echo imaging sequence (2 × 2 × 2 mm^3^ voxel size; 65 slices; echo time 1/echo time 2/repetition time = 5.19/7.65/655 msec) to account for distortions caused by field inhomogeneities.

### MRI analysis

All images were analyzed using tools from the FMRIB Software Library (FSL; FMRIB, Oxford, UK; www.fsl.fmrib.ox.ac.uk/fsl/fslwiki/).

### DTI general pre-processing

Each subject’s DTI scans were corrected for head motion and eddy currents and then brain-extracted to remove any non-brain voxels. To correct for B_0_ inhomogeneities and unwarp scans, field map correction was performed with FUGUE. FA, mean diffusivity (MD), axial diffusivity (AD, eigenvector L1), and L2 and L3 maps were created using DTIFIT by applying a diffusion tensor model to each voxel. RD maps were created by averaging the L2 and L3 maps (RD = (L2 + L3)/2).

### DTI tract-based spatial statistics (TBSS) preprocessing

All individual FA images of all subjects were nonlinearly registered to a standard FA template (http://fsl.fmrib.ox.ac.uk/fsl/fslwiki/FMRIB58_FA), and then averaged to create a study-specific template to which each subject’s FA map was then nonlinearly registered. Next, the mean FA image was created and thinned to create a mean FA skeleton, which represents the centers of all tracts common to the group. Each subject’s aligned FA data were then projected onto this skeleton. The same operations that were used to register the individual FA images to the study-specific template and project FA values onto the mean FA skeleton were subsequently applied to the individual MD, L1, and RD images.

### Statistical analyses

The Juelich Histological Atlas was used to produce masks of the left and right CST, callosal body (CC), and superior longitudinal fascicle (SLF) in Montreal Neurological Institute (MNI) standard space. The CST and CC are obvious a priori region-of-interest (ROI) choices in ALS. The SLF was chosen based on previously published studies reporting involvement of extra-motor tracts in ALS. For the TBSS analysis, where the final ROI results from the intersection of the atlas-based masks and the mean FA skeleton mask, the left and right CST and CC masks were thresholded at “45” and the left and right SLF masks thresholded at “10” (as described previously in Menke et al.^11^).

Voxel-wise General Linear Model (GLM) was applied using permutation-based non-parametric testing to regress CSF and serum NfL concentrations with the pre-processed TBSS images separately in patients and controls (controlling for age by inclusion as a covariate of no interest). Results were considered significant for *P* < 0.05, after correction for multiple comparisons (family wise error, FWE) within each region of interest, using the threshold-free cluster enhancement (TFCE) approach.^13^

CSF and serum NfL concentrations were separately correlated with age in both groups, and with UMN score and progression rate in the ALS group (Spearman correlation implemented in IBM SPSS Statistics 21; IBM Corporation, Armonk, New York, US).

## Results

### Participants

Twenty-five patients and 17 healthy, age-matched controls with CSF NfL levels and MRI data obtained on the same day were available for analysis (from a total of 72 patients and 35 controls enrolled in the wider BioMOx study). Paired serum samples and MRI data were available for a larger cohort of 40 patients and 25 controls. Participant features are summarized in Table1.

**Table 1:** Summary of study participant features (mean values and standard deviation in parentheses)

Group		Age (years)	Sex	Site of symptom onset	Disease duration (months)	UMN score	ALSFRS-R	Rate of progression
CSF	ALS	61.5 (11.2)	7F:18M	Bulbar 6	34.8 (29.7)	10.3 (3.8)	35.2 (5.5)	0.68 (0.78)
				R limb 11				
				L limb 8				
	Controls	54.3 (11.3)	10F:7M	n/a	n/a	n/a	n/a	n/a
Serum	ALS	60.2 (11.5)	13F:27M	Bulbar 7	39.8 (31.1)	9.8 (4.0)	34.8 (5.6)	0.56 (0.65)
				R limb 19				
				L limb 13				
				Both legs 1				
	Controls	52.4 (12.6)	15F:10M	n/a	n/a	n/a	n/a	n/a

UMN, upper motor neuron; ALSFRS-R, revised ALS Functional Rating Scale; CSF, cerebrospinal fluid; ALS, amyotrophic lateral sclerosis; F, female; M, male.

### CSF and serum NfL concentrations

In both groups, CSF and serum NfL concentrations were significantly correlated (patients: *ρ* = 0.642, *P* = 0.001; controls: *ρ* = 0.677, *P* = 0.004).

**Figure 1:**
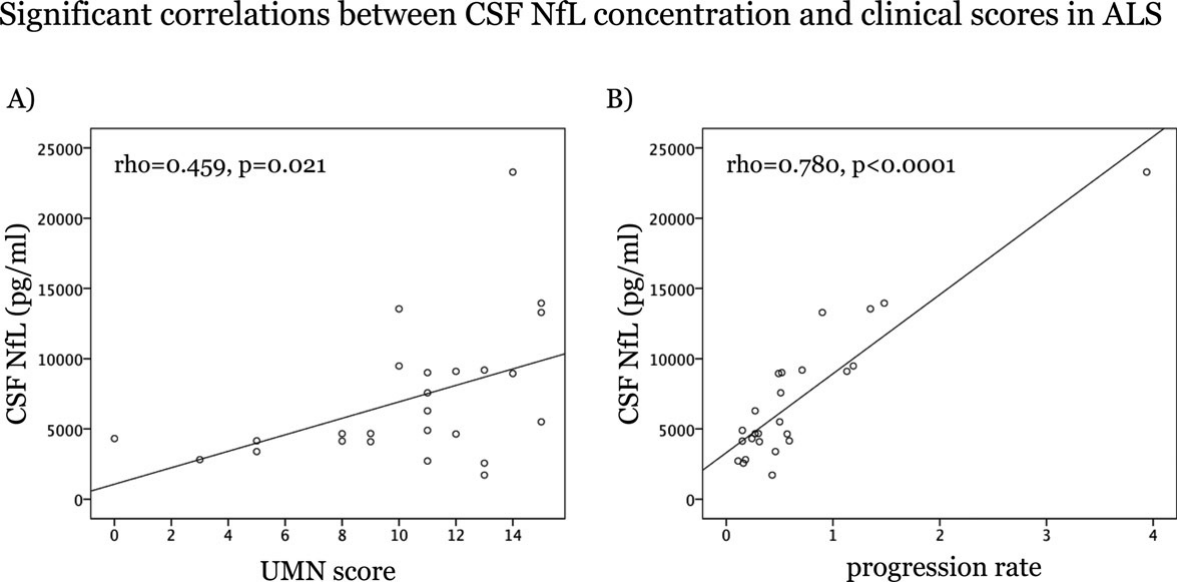
Scatterplots of CSF NfL concentration and UMN score (A) and progression rate (B) in the ALS patients. CSF, cerebrospinal fluid; NfL, neurofilament light chain protein; UMN, upper motor neuron; ALS, amyotrophic lateral sclerosis.

Mean CSF NfL levels were significantly higher in patients (7118 ± 4879 pg/mL) versus controls (663 ± 464 pg/mL; *P* < 0.0001, Independent-Samples Mann–Whitney *U* Test). CSF NfL concentration was positively correlated with age (*ρ* = 0.800, *P* < 0.0001) in controls, but not in patients. In patients, CSF NfL levels correlated positively with UMN score (*ρ* = 0.459, *P* = 0.021), and progression rate (*ρ* = 0.780, *P* < 0.0001) (Fig.1).

As in the overall sample patients and controls differed significantly with respect to age (*P* = 0.048) and gender distribution, we also compared CSF NfL levels between a closely age-matched subset of 15 patients (seven female, eight male; mean age = 56 ± 10 years) and 15 controls (eight female, seven male; mean age = 55 ± 12 years; *P* = 0.909). The results of the group comparison remained qualitatively unchanged for this smaller cohort; with significantly higher mean CSF NfL level in patients (7446 ± 5392 pg/mL) versus controls (700 ± 482 pg/mL; *P* < 0.0001, Independent-Samples Mann–Whitney *U* Test).

Mean serum NfL level was also significantly higher in patients (97 ± 90 pg/mL) versus controls (30 ± 31 pg/mL; *P* < 0.0001, Independent-Samples Mann–Whitney *U* Test). Serum NfL concentration was also positively correlated with age (*ρ* = 0.643, *P* = 0.001) in controls, and correlated positively with UMN score (*ρ* = 0.341, *P* = 0.031), and progression rate (*ρ* = 0.564, *P* < 0.0001) in patients.

### CSF and serum NfL correlation with DTI measures

**Figure 2:**
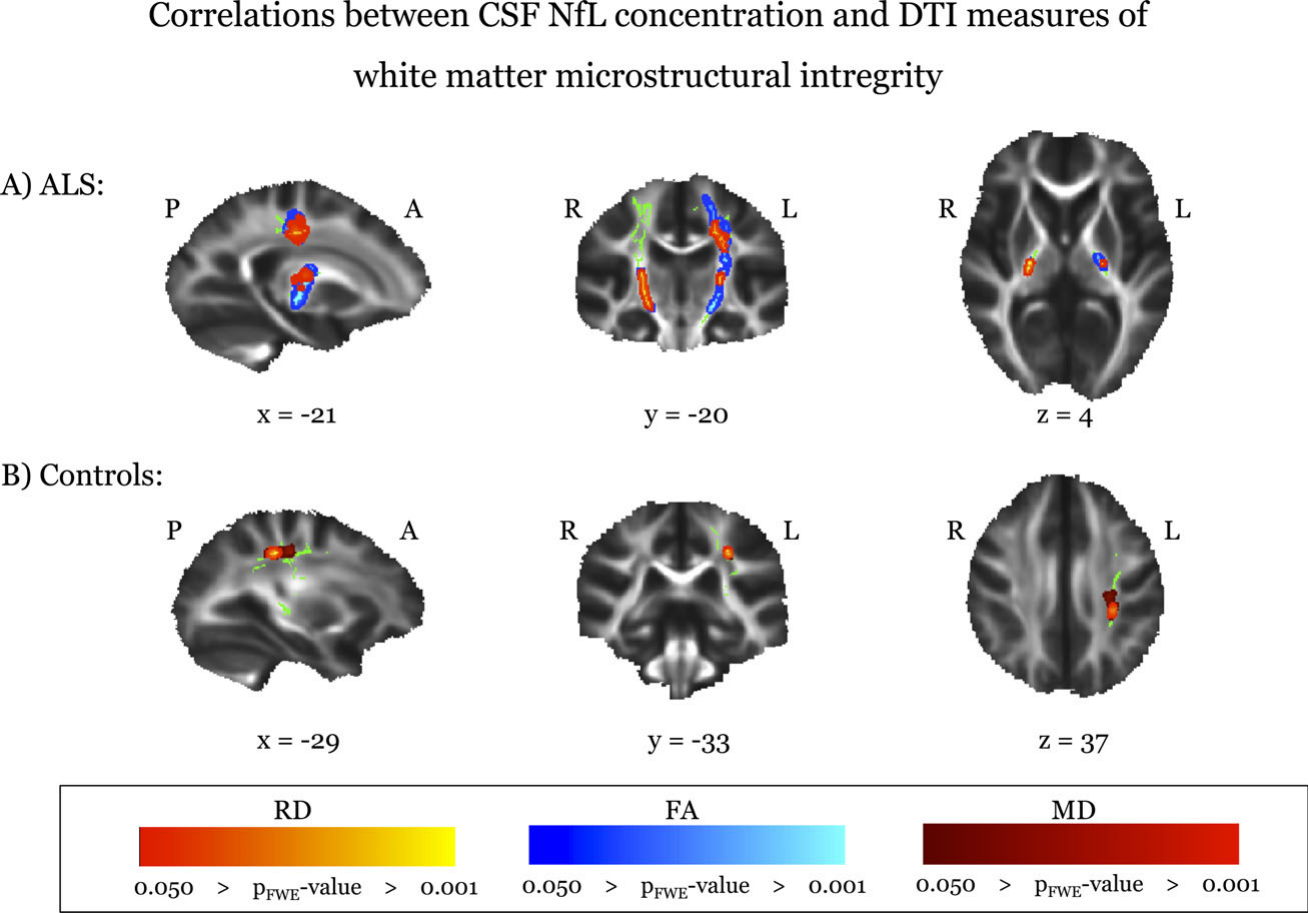
Significant TBSS correlations between CSF NfL concentration and DTI measures in the ALS (A) and in the control (B) group. TBSS results are overlaid onto the intersection of the skeleton with the respective ROI (green) and the group’s mean FA image (greyscale). TBSS, tract-based spatial statistics; CSF, cerebrospinal fluid; NfL, neurofilament light chain protein; DTI, diffusion tensor imaging; ALS, amyotrophic lateral sclerosis; ROI, region-of-interest; RD, radial diffusivity; FA, fractional anisotropy; MD, mean diffusivity; P, posterior; A, anterior; R, right; L, left. *x*, *y*, *z* = MNI coordinates.

**Figure 3:**
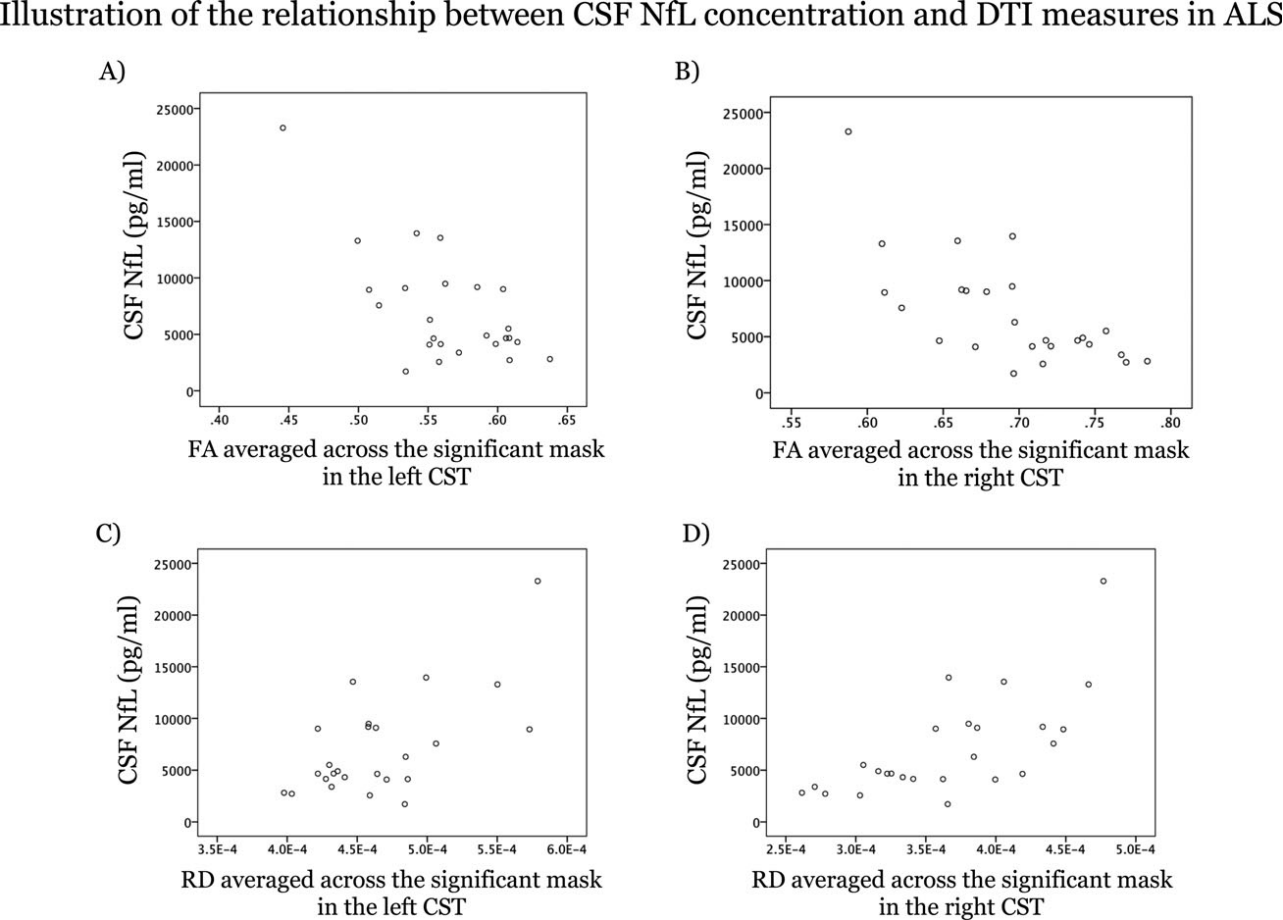
Scatterplots illustrating the relationship between CSF NfL concentration and DTI measures in the patient group: FA in the left (A) and right (B) CST; RD in the left (C) and right (D) CST. CSF, cerebrospinal fluid; NfL, neurofilament light chain protein; DTI, diffusion tensor imaging; CST, corticospinal tract; FA, fractional anisotropy; RD, radial diffusivity.

TBSS analysis revealed significant (*P*_FWE_ < 0.05, corrected for age) negative correlation between NfL measures and FA (co-localized with positive RD correlation) in both CSTs in ALS patients (Fig.2A). Similarly in the larger serum cohort, NfL levels correlated negatively with FA (and positively with RD) in both CSTs (Fig. S1A). In addition, serum NfL levels correlated significantly with axial diffusivity in a small region in the right SLF in patients (Fig. S1B). In the healthy control group, only a positive correlation between CSF NfL (but not serum NfL) concentration and RD for a small region in the left SLF was observed (Fig.2B). Figure3A–D depicts the relationship between CSF NfL concentrations and the respective DTI measure for the significant TBSS correlations in the patient group (DTI indices were averaged across the entire region of the skeleton in which a significant correlation was observed (“significant mask”) to generate a single value for illustrative purposes).

## Discussion

### CSF NfL and ALS pathophysiology

The significantly elevated CSF NfL concentrations in the ALS patients are consistent with previous studies that reported elevated CSF NfL levels in ALS compared with healthy and disease controls.^2^–^4^ CSF NfL concentrations have also been noted to be higher in ALS patients with faster progression rates.^5^,^6^ In ALS, it is established that there is a selective loss of large myelinated axons in the CST of ALS patients.^14^ Markedly elevated CSF NfL levels in ALS may be explained by the higher content of axonal proteins in motor neurons compared to other neuronal populations.^15^ A strong DTI white matter signature involving the CSTs has been consistently identified in ALS,^9^,^16^ with FA in the CST, in particular the posterior limb of the internal capsule (PLIC), linked to progression rate.^10^,^17^ While the patients and healthy controls included in the present studies were not matched for age or gender, group comparisons of NfL levels for an age-matched subset of participants led to qualitatively similar results, and we are not aware of any reports highlighting gender differences in CSF NfL concentrations. Taken together, we believe our observations reflect the release of NfL into the CSF from the degenerating CSTs in particular due to the abundance of neurofilaments in large axons and the highly soluble nature of NfL.^18^ There is undoubtedly a contribution from the concurrent degeneration of the CST within the spinal cord in ALS, which was not captured by the DTI in this study. The number of patients and controls with paired CSF NfL and MRI data in the BioMOx cohort was limited by the practical challenges of obtaining combined markers in disabled patients and the invasive nature of CSF extraction in terms of recruitment. However, the qualitatively similar results obtained with serum NfL from a larger patient group support the conclusions drawn, despite being potentially influenced by degeneration of peripheral motor axons.

### CSF NfL in other disorders

CSF NfL is not a specific marker for ALS. Elevated levels have been reported in spinal cord injury^19^ as well as other neurodegenerative disorders,^12^ specifically FTD.^20^,^21^, Alzheimer’s disease (AD),^22^ and Parkinsonian disorders.^23^ Few studies have attempted to link NfL and MRI measures, however. Higher concentrations of NfL were related to more pronounced white matter changes based on T2- and proton density-weighted scans in subjects with AD, subcortical vascular dementia and healthy controls.^24^ Baseline CSF NfL concentration predicted localized FA differences in the middle temporal gyrus white matter in adults at risk for AD.^25^ Only antibodies to NfL (rather than measured NfL levels) were linked to MRI markers of axonal loss in multiple sclerosis (MS).^26^

### DTI and CSF NfL

The core cerebral white matter DTI signature in ALS involves the CSTs, confirmed across many patient cohorts.^27^ We observed CSF NfL correlations in the CSTs for FA, and RD in particular, but not axial diffusivity (L1). RD may be sensitive to Wallerian-type myelin degeneration,^28^ as noted in ALS. Independent MRI or CSF measures of myelin content in ALS would be desirable to unravel the precise origins of NfL in the CSF. We have previously studied myelin integrity using a novel MRI sequence sensitive to water pools within myelin and intra- and extra-cellular spaces, known as Multi-component Driven Equilibrium Single Pulse Observation of T1 and T2 (mcDESPOT).^29^ However there were insufficient BioMOx subjects with both mcDESPOT and CSF or serum NfL measurements to carry out an analysis of their relationship.

Lower CSF NfL levels have been noted in patients with ALS linked to superoxide dismutase (*SOD1*) mutations compared to cases with wild-type *SOD1*.^30^ Independent studies in a group of consistently slowly progressive familial ALS patients homozygous for the *SOD1* “D90A” mutation showed a curious relative sparing (in DTI terms) of CST involvement compared with sporadic ALS patients matched for disability and clinical UMN involvement.^31^ These two findings suggest that CSF NfL levels (and DTI measures) may be significantly influenced by genotype. Our study population was uniformly apparently sporadic, but future studies should also consider potential genotype effects.

In conclusion, elevated CSF NfL concentrations (and the highly correlated serum levels) found in ALS appear to be related to white matter damage within the CSTs as assessed by metrics derived from DTI. This provides further support for NfL as a meaningful biomarker in ALS. Prospective studies in those suspected to have ALS, in established ALS mimic disorders, and in those presymptomatic individuals carrying genetic mutations placing them at high risk of ALS, are now underway to fully explore the biomarker potential of combined neurochemical and MRI measures.

## Supporting Information

**Figure S1.** Significant TBSS correlations between serum NfL concentration and DTI measures in the ALS group: (A) CSTs, (B) right SLF. TBSS results are overlaid onto the intersection of the skeleton with the respective ROI, green) and the group’s mean FA image (greyscale). MD, mean diffusivity; FA, fractional anisotropy; L1, axial diffusivity; P, posterior; A, anterior; R, right; L, left. *x*, *y*, *z* = MNI coordinates.
